# Hypodermic Injections

**Published:** 1868-04

**Authors:** A. S. Maxwell

**Affiliations:** Chairman


Reported to the Iowa State Medical Society, at its session in Davenport, May, 1867.

BY A. S. MAXWELL, M. D., CHAIRMAN.

THE various means that have been in use to introduce medicines into the system by other routes than the mouth, are well known to all present. The only query that now appears to us is, "that we should have been so long without this additional aid"—*the Hypodermic Syringe*—after knowing as much as was generally known about the absorbing power of the cellular tissue. We are only too sorry to have to record that our knowledge on this subject has been mainly obtained at the sacrifice of many valuable lives; for, it has long been known that, if the virus of any poisonous reptile or rabid animal was introduced beneath the *cutis vera*, very dangerous results might be anticipated, so much so that *we*, "humbling as it may *appear*," must confess that *medical men* have, in most cases, utterly failed to do more than act the part of supplicants to this the most formidable of all diseases, instead of being able to wrest from its grasp its unhappy victims, and bid defiance to its insidious assaults.

Since then, we have learned of ourselves, and been forced to heed the admonitions of the lower animals, in this, that any substance which is capable of being absorbed, if introduced beneath the cuticle, we may expect from it prompt and immediate action. This leads us to inquire whether there is any difference between absorption as compared with the depth introduced. I have not noticed the point commented on by any one; yet, my own belief is, that a much more prompt and immediate result is obtained when the medicament is introduced as deeply as possible.

Much more time might be occupied on this preliminary part of our subject; but for the present we pass it, hoping that in the future we will hear a much more elaborate article on "*The celular tissue*—its relation to medicine."

We would now take pleasure in giving you a minute statement as to how and by whom this instrument was first introduced to the notice of the profession; but in the meagre accounts given us we cannot attempt to decide between the claims of "*the host*" who represent themselves to be its inventors. This is one of the many inventions that we are compelled to refer to the *Censors* of future generations. Nor would it be a profitable investigation to attempt to learn who utilized it, since it appears that not only in different countries but in various parts of our own country many persons began to use it so near the same time that to accord any one precedence would be doing grave injustice to perhaps hundreds of others; hence we leave its origin and early history to abler commentators, and now notice its claims as a therapeutie agent.

Pain, the common ill of mankind—tersely defined by one to be "the prayer of the nerves for healthy blood"—demanded that there be some means devised by which it might be controlled until the craving nerves were supplied by other and slower means. How well this demand has been answered prior to the introduction of this instrument each one can answer; but armed with it we can fully control the lamentable and bewailing cry so often heard by each one of us, "Oh, Doctor, for God's sake relieve me!" and in thus relieving pain so often only a symptom, yet so formidable a foe wherever present, as to demand of us our undivided efforts, we have done much to promote the action of other remedies.

We will next momentarily compare it with the long established means of treatment. You all well know that medicines taken into the stomach pass by way of the *portal system* to the circulation, hence the condition of the digestive apparatus and circulation have much to do with the results obtained. while by the mode now being described it passes direct into the circulation and produces an instantaneous effect.

The difficulties and disadvantages attendant on the giving of medicines by the rectum are of such a character as to very much interfere with this as being a popular and efficient means of treatment; besides, too, the rectum being to a great extent controlled by the reflex system, renders the action of medicines directed to the cerebro-spinal system as almost wholly powerless.

To accomplish the endermic method we must often do directly the opposite of that which the case demands—as blistering, &c., causing a local irritation, to say the least of it, both unpleasant and troublesome—besides the uncertainty that must forever exist as regards the amount that will be absorbed.

Hypodermically, we know that we will have an effect equal to the amount given, whilst we can accurately determine how much is given.

When this treatment was first began it was wholly applied to relieve cases of neuralgia of a strictly local character, but steadily its field of usefulness increased, until now it is considered an invaluable aid in the treatment of rheumatism, paralysis, gout, chorea, tetanus, neuralgia, malarial fever, and many others; and yet we think it has not reached its maximum height of usefulness.

The immense variety of forms of disease in which this is applicable make it patent to every mind that to give a full detail of medicines to be employed would not only be impracticable, but nearly impossible. The following system we have observed and believe to be practicable:

That larger doses may be given in chronic cases than in acute.

That males should have larger doses than females.

That first applications should always be of minimum size; and that the dose to be given should be about one-half as much as directed by the mouth.

Great difference of opinion exists as to the point of application. The weight of authority is perhaps favorable in localized affections to the application being made at or near the point diseased; but as a general rule I believe the arm to be the most eligible and best suited point of application.

If you will bear with me a little longer, I will narrate the interesting points of a few cases that have occurred in my own practice.

The first time I saw this instrument used was by Dr. Langer, of this city, in 1857. He, I think, tried it in three cases of neuralgia of different parts—the exact specifications I do not remember. Suffice it to say that the treatment was so satisfactory that I was induced immediately to experiment with it.

*Case First.*—Male, aged 60; sanguine temperament, healthy constitution, but had been a martyr to facial neuralgia for several years. I injected a solution of morphine near affected point, with the result of first treatment palliating the paroxysmal pain; the second entirely relieving him of his troublesome affection.

*Case Second.*—Male, aged 26; recurrent intermittent fever. In connection with constitutional treatment, I injected Quinia. Sulp. gr. ij., repeated three times at variable intervals.

*Case Third.*—Lady, aged 35; plethoric habit; constitution vigorous and healthy; attacked suddenly by puerperal mania. Morphia Sulph. gr. ½, frequently repeated, was used with decidedly good results.

In lumbago, sciatica, and rheumatism, I have used many of the anti-spasmodic and narcotic medicines, but feel constrained to report my belief that in these the effect is only palliative, but yet useful as addendums to constitutional treatment.

*Case Fourth.*—A private of the 8th infantry Iowa volunteers, aged 25; robust and muscular; but after long marches and great exposure, was suddenly attacked with complete paralysis of lower extremities, and partial of the upper. After treatment in the regimental hospital for some weeks, he was sent North, where I treated him principally by subcutaneous injections and tonics. Within three weeks he returned to active duty with his regiment. In April, 1864, he was again attacked with same disease, and was again treated by me in the same manner with satisfactory results.

*Case Fifth.*—Private of 37th regiment Iowa volunteers, aged about 55. After being considerably reduced by chronic diarrhœa, was taken with paralysis of the right leg and arm. The treatment pursued was the same as the last quoted case, with results less prompt, but nearly as satisfactory.

I have yet to report two cases that were of interest to me. Mr. and Mrs. ———, of Vicksburg, while traveling by steamboat up the Mississippi, were attacked with congestive chills. In connection with the constitutional treatment which each one of them received, I gave him hypodermically Atrophia gr. $\frac{1}{35}$, repeated twice. His recovery was prompt, decided and satisfactory; while she recovered only after a long and serious illness. The character of the reaction in this case was such as to impress me with the fact of its being highly beneficial in cholera; but I have not had a favorable opportunity by which to test its merits. If any one present has had an experience with it in this disease it would afford me much pleasure to hear from them.

In conclusion allow me to suggest that with all this long array of diseases and remedies, and the great benefit derived from subcutaneous injections, I do not wish to be understood to mean that wherever pain exists, all that is necessary is to use this means of medication. With this, as with all other potent remedial agents—powerful for good or evil, a fitly chosen case, with proper applications, good results may be expected; but an indiscriminate use of it will not only disgust all parties concerned, but will also materially paralyze its future prospects.

---

THE use of the Hypodermic Syringe has become a very popular mode by which medicines are introduced into the system, and the success attending its use in very many cases is entirely satisfactory. Its use in the introduction of acetic acid in various forms of cancer has created much interest within the last year or two; but the success attending it is not meeting the expectation of its advocates.

